# Extremely High Brightness from Polymer-Encapsulated Quantum Dots for Two-photon Cellular and Deep-tissue Imaging

**DOI:** 10.1038/srep09908

**Published:** 2015-04-24

**Authors:** Yanyan Fan, Helin Liu, Rongcheng Han, Lu Huang, Hao Shi, Yinlin Sha, Yuqiang Jiang

**Affiliations:** 1 State Key Laboratory of Molecular Developmental Biology, Institute of Genetics and Developmental Biology, Chinese Academy of Sciences, Beijing 100101, China; 2 Department of Biophysics, School of Basic Medical Sciences and Biomed-X Center, Peking University, Beijing 100191, China; 3 Institute of Opto-electronic Materials and Technology, South China Normal University, Guangzhou 510631, China

## Abstract

Materials possessing high two photon absorption (TPA) are highly desirable for a range of fields, such as three-dimensional data storage, TP microscopy (TPM) and photodynamic therapy (PDT). Specifically, for TPM, high TP excitation (TPE) brightness (σ × ϕ, where σ is TPA cross-sections and ϕ is fluorescence quantum yield), excellent photostability and minimal cytotoxicity are highly desirable. However, when TPA materials are transferred to aqueous media through molecule engineering or nanoparticle formulation, they usually suffer from the severely decrease of quantum yield (QY). Here, we report a convenient and efficient method for preparing polymer-encapsulated quantum dots (P-QD). Interestingly, the QY was considerably enhanced from original 0.33 (QDs in THF) to 0.84 (P-QD in water). This dramatic enhancement in QY is mainly from the efficiently blocking nonradiative decay pathway from the surface trap states, according to the fluorescence decay lifetimes analysis. The P-QD exhibits extremely high brightness (σ × ϕ up to 6.2 × 10^6^ GM), high photostability, excellent colloidal stability and minimal cytotoxicity. High quality cellular TP imaging with high signal-to-background ratio (> 100) and tissue imaging with a penetration depth of 2200 μm have been achieved with P-QD as probe.

Two photon absorption (TPA) materials have drawn increasing interest because of their wide ranges of prominent potential applications, such as three-dimensional data storage^1^,^2^, TP microscopy (TPM)^3^ and photodynamic therapy (PDT)^4^. In compared with one-photon microscopy, TPM can provide several advantages, including intrinsic three-dimensional resolution, greater imaging penetration depth and reduced overall phototoxicity. For applications in TPM, TPA material should possess high TP excitation (TPE) brightness, superior water-dispersiblity, excellent chemical stability and photostability, as well as minimum cytotoxicity. Different kinds of TPA materials have been developed toward the creation of TP bioprobes and some of them have been successfully used for TP biomedical imaging^5^,^6^. Despite these previous achievements, a major limitation of TPM is the lack of available probes with high TPE brightness because most of them have very small TPA cross-sections (σ), typically σ < 50 GM (1 GM = 10^−50^ cm^4^ s photon^−1^)^7^, and/or low quantum yield (QY, ϕ). To develop novel highly bright TP probes is of importance for TPM, especially in improving imaging sensitivity and depth.

To develop probes with enhanced TPE brightness, one straightforward way is to synthesize new materials with high TPA cross-sections via molecule design or engineering^8^,^9^. Meanwhile, incorporating these available fluorescence materials into a nano-sized particle is another feasible and efficient strategy^10^,^11^,^12^. However, one limitation of encapsulation strategy is typically a decrease in QY, and hence severely decreased fluorescence brightness of the material. Till now, only a few fluorophores, such as aggregation induced emission (AIE) dyes that show no emission in dilute solutions but are brightly fluorescent upon concentration or solidification^13^, have been successfully used to prepare bright TP nanoparticles via nanocapsulation strategy^10^,^14^,^15^. Recently, based on europium complex Eu(tta)_3_dpbt^16^ (tta = thenoyltrifluoroacetonato, dpbt = 2-(N,N-diethylanilin-4-yl)-4,6-bis-(3,5-dimethylpyrazol-1-yl)-1,3,5-triazine) and its derivatives^17^,^18^, we have developed a series of highly bright TP probes with nanocapsulation strategy. High TPE brightness (σ × ϕ ~ 10^5^ GM at 830 nm) and high quality live cell TP imaging have been achieved^19^,^20^,^21^.

Quantum dots (QDs), also known as semiconductor nanocrystals, have been demonstated to possess extraordinarily high TPA cross-section, σ ~ 10^4^ GM^22^, which is orders of magnitude higher than most organic dye molecules. As an excellent TPA materials, QDs have been successfully applied to TP bioimaging^23^,^24^,^25^,^26^. However, the TP imaging depth was not well documented^22^. Besides, the biocompatibility is another major issue, as QDs mostly contain cadmium or other heavy metals. In recent years, polymers with excellent biocompatibility have been successfully and widely employed to modify QDs surface^27^ and these polymer-encapsulated QDs are found to be essentially nontoxic to cells and animals^28^,^29^,^30^. One of the costs of such nanocapsulation strategy is usually a decrease in QY, and hence severely reduced fluorescent brightness of the material^31^,^32^,^33^,^34^. Therefore, the development of QDs-based TP probes without degrading the original superior optical properties, and meanwhile with excellent photostability and minimal cytotoxicity, is techinally challenging, but highly desirable for pratical TPM.

Herein, a novel polymer-encapsulated QDs (P-QD) was prepared via co-precipitation-assembly process. The P-QD is characterized by encapsulation of CdSeS/ZnS QDs in the matrix material of poly(methyl methacrylate-co-methacrylic acid) (PMMA-co-MAA). The QY was considerably enhanced from 0.33 (original QDs in THF) to 0.84 (P-QD in water). And the P-QD exhibited impressive high TPE brightness σ × ϕ, up to 6.2 × 10^6^ GM (1 GM = 10^−50^ cm^4^ sphoton^−1^ particle^−1^) at 800 nm. Besides superior TP luminescence properties, owing to rationally design and carefully choosing the proper matrix materials, the P-QD was also endowed with high photostability, excellent colloidal stability and minimal cytotoxicity. And high quality cellular TP imaging with high signal-to-background ratio (SBR, > 100) and tissue imaging with a penetration depth of 2200 μm were successfully achieved with P-QD as probe.

The P-QD was synthesized by our previously developed co-precipitation-assembly method^19^. The prepared CdSeS/ZnS QDs were passivated by organic ligands (i.e., tri-n-octylamine and oil acid) and thus were well-dispersed in organic solvents, but not in water. When a THF solution containing QDs and PMMA-co-MAA was added into the aqueous solution, co-precipitation and assembly of these compounds occurred to form the present colloidal nanoparticles (Figure 1), accompanied by the encapsulation of QDs in the hydrophobic cores and exposing a part of the carboxyl groups to water^19^.

## Results and Discussions

**Figure 2:**
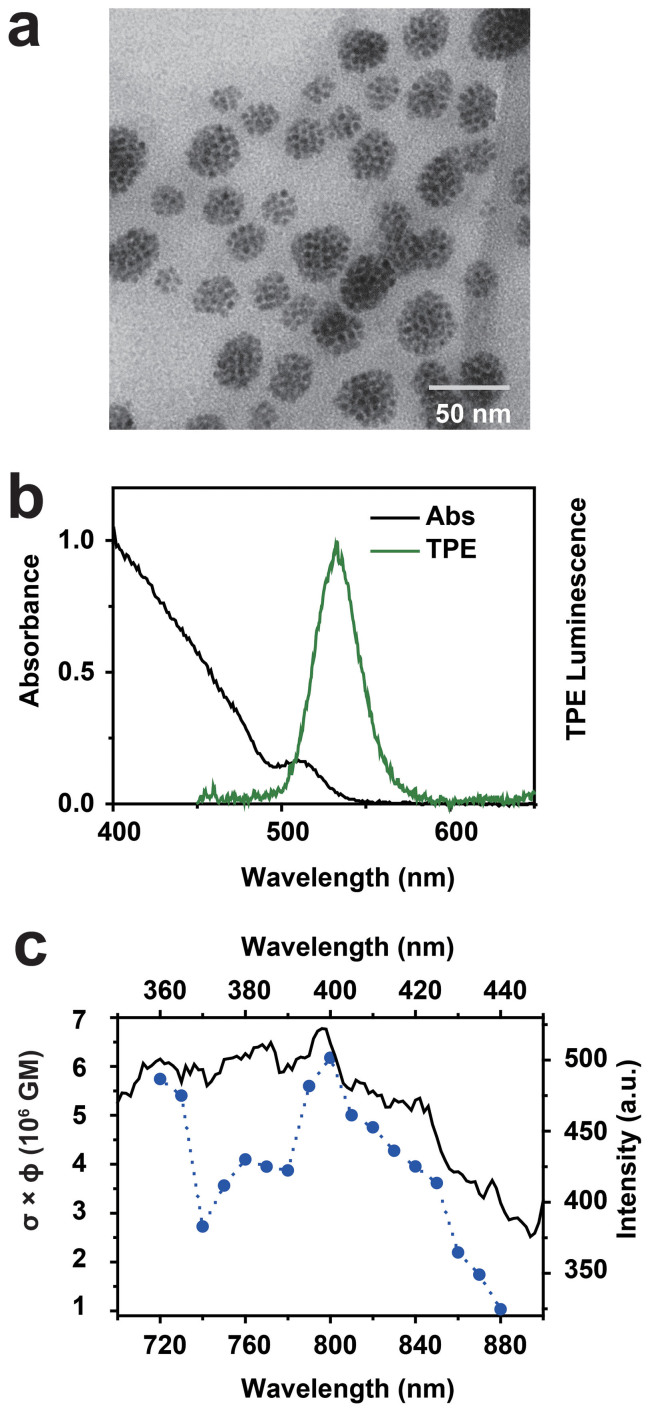
(a) Transmission electron microscopy (TEM) image of P-QD. (b) UV-vis absorption and two-photon excitation (TPE) photoluminescence spectra (*λ*_ex _ = 800 nm) of P-QD in water. (c) TPE brightness (σ × ϕ) and one-photon excitation spectrum (*λ*_em _ = 530 nm) (solid line) for the P-QD solution (1 GM = 10^−50^ cm^4^ sphoton^−1^ particle^−1^). Colloidal P-QD dispersed in water were used in these measurements.

The transmission electron microscopy (TEM) image, shown in Figure 2a, demonstrates that multi-QDs were embedded in PMMA-co-MAA nanosphere. Notably these QDs are highly dispersed within P-QD and no apparent aggregation was observed and thus efficiently decrease the fluorescence self-quenching. Our synthesized P-QD exhibited an average diameter of 27.1 ± 5.5 nm (N = 313, Figure S1a). Considering the diameter of original CdSeS/ZnS QDs (~ 4 nm, Figure S2), the number of QDs within the polymer nanosphere was estimated to be maximum about 300 per single P-QD.

Dynamic light scattering data indicated that the hydrodynamic diameters of P-QD had a narrow distribution around 76 nm (Figure S1b). On the other hand, the zeta potential of the P-QD dispersed in deionized water was measured to be −24.4 ± 0.7 mV, which can be ascribed to the ionization of the surface carboxyl groups. Due to this high surface zeta potential, no precipitation or agglomeration was observed after the prepared colloidal solutions were stored under ambient conditions for at least 6 months, suggesting the excellent colloidal dispersion stability of P-QD.

The as-prepared aqueous solution of P-QD exhibited bright green emission under UV excitation (Figure S3a). And further spectroscopic measurements revealed that P-QD in aqueous solution presented narrow symmetrical emission, only ~ 1 nm red-shift of the original PL emission (Figure S3b). This slight red-shift may be related to solvent effects and/or the presence of surface charges^34^,^35^. Figure 2b shows the optical absorption of P-QD in aqueous solution. Compare to that of the original QDs, there is no apparent absorption peak shift observed in P-QD. These results demonstrate that the distinctive absorption and PL emission profiles of QDs are well preserved after encapsulation process.

Figure 2b also shows the emission spectrum of P-QD under 800 nm femtosecond laser irradiation. The emission spectra under various laser power were further performed and a log-log plot of the PL intensity versus laser power gave a slope of 1.94 ± 0.02, as shown in Figure S3c. This value unambiguously indicates that the excitation power dependence is quadratic and, therefore, confirms the TPE origin of the luminescence. And the maximum emission wavelength is similar to that of original QDs under one-photon excitation (OPE), while the bandwidth is slightly narrower than that of the OPE PL spectrum (Figure S3d).

It should be noted that the QY was considerably enhanced from 0.33 (original QDs in THF) to 0.84 (P-QD in water). This dramatic enhancement in QY is quite reproducible, which was confirmed in studies of different samples (Table S1 and Figure S4). This QY enhancement effect in QDs via surface passivation/modifications has been reported by others^36^,^37^. Although different mechanisms have been proposed^38^, the exact mechanism behind this enhancement effect is still not fully understood. To explore the mechanism behind our QY enhancement phenomena, the fluorescence decay lifetimes of original QDs and P-QD were measured. These kinetics decay traces were fitted by double exponential decay functions, typically displaying a two-component decay (*τ*_1_ ~ 4 ns and *τ*_2_ ~ 20 ns, Figure S5). Moreover the fast component increased from original 25.6% (QDs in THF) to 31.6% (P-QD in water), and continuously increased up to 46.3% with increasing the fraction of QDs. As discussion in Supporting Information, the QY improvement of P-QD is mainly ascribed to the efficiently blocking nonradiative decays pathway from the surface trap states via surface modification.

According to the method described in the literature^39^ (Supporting Information), the concentration of QDs was determined to be 1.4 × 10^−7^ M. And the TPE brightness σ × ϕ was determined with fluorescein in basic aqueous solution as the reference^40^. For our P-QD system, it was found that the maximum TPE brightness occurred near 800 nm with σ × ϕ value of 6.2 × 10^6^ GM per particle (Figure 2c and Table S2). This σ × ϕ value is 2 orders of magnitude higher than that of CdSe/ZnS QDs determined by a similar method^22^. The following factors may be related to this giant TPE brightness. First of all, the QY of P-QD was determined to be as high as 0.84, about 2.5 fold higher than that of original QDs. Secondly, this giant σ × ϕ value may be related to the local concentration effect. As the diameter of P-QD and original QDs is ~ 27 nm and ~ 4 nm, a large number of (maximum about 300) QDs encapsulating into a P-QD can be expected and thus the local concentration is significantly enhanced, resulting in the huge TPA cross-section per particle. Finally, most of the adverse effect of quenching are greatly reduced and even blocked via varying experimental conditions. These factors together contribute to this extremely high TPE brightness and this giant σ × ϕ value endows the possibility of P-QDs use in TPM.

For biological applications, the biocompatibility of a TP probe is a major concern, especially for heavy-metal-containing QDs. Cytotoxicity tests were further performed with HeLa and L929 cells by the methyl thiazolyl tetrazolium (MTT) assay. The living cells were exposed to buffer solutions with different concentrations of P-QD for 24 h, and the percentages of the viable cells were quantified. As shown in Figure S6, no apparent cytotoxicity was observed in HeLa and L929 cells even when the concentration of QDs was increased up to 600 nM. The high biocompatibility of PMMA-co-MAA and the efficient shielding of QDs by the matrix from surrounding may be a cause of the low cytotoxicity of P-QD to the cells.

**Figure 3:**
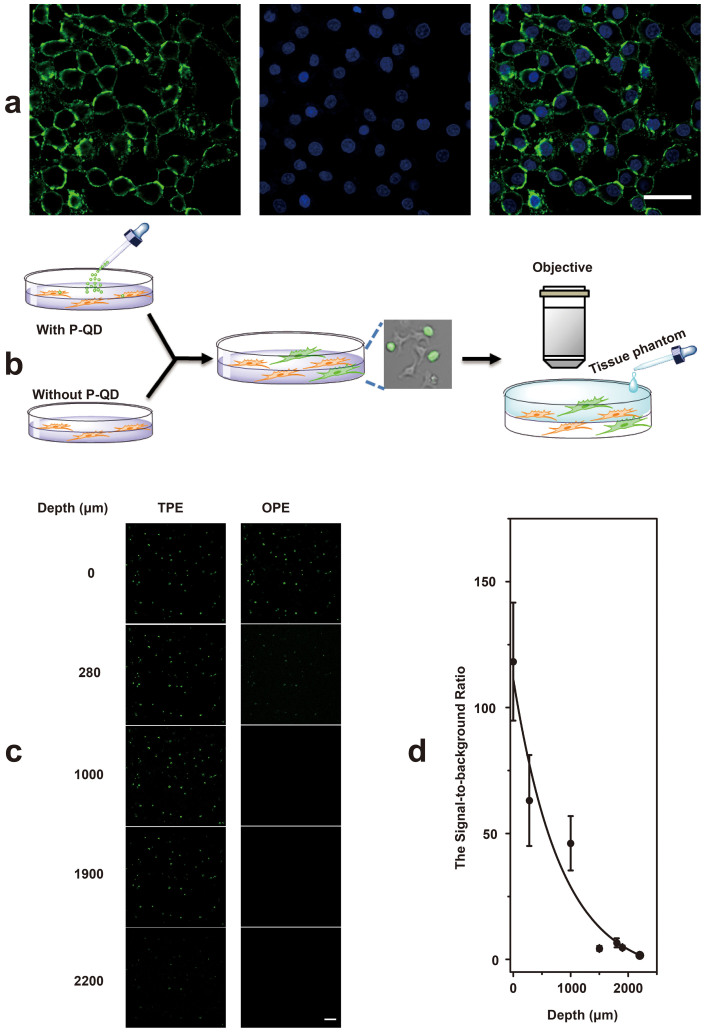
(a) Two-photon imaging of Human hepatocellular liver carcinoma cell line (HepG2) after treated with P-QD for 2  h, 800 nm femtosecond laseras excitation. Left: P-QD; middle: Hoechst; right: overlay. Note that these images were acquired by spectral imaging coupled with image analysis using linear unmixing. Scale bar: 50 μm. (b) Schematic of the procedure used for TPM of P-QD in tissue phantom with different thickness. The depth of tissue phantom was varied by adding different amounts of Intralipid. (c) Imaging depth of P-QD in tissue phantom under 800 nm femtosecond laser excitation. For comparison, the corresponding pictures under one-photon excitation (OPE) are also shown. Scale bar: 100 μm. (d) The signal-to-background ratio (SBR) as a function of depth. SBR was defined as the ratio of (signal_with P-QD _− signal_without P-QD_) to signal_without P-QD_.

To demonstrate the potential utility of P-QD for TPM, *in vitro* cell imaging was performed by incubating HepG2 cells with P-QD, as shown in Figure 3a. Bright TPE emission signal was detected from P-QD treated HepG2 cells. And these signals were further confirmed by the emission profiles (Figure S7). Notably the SBR in P-QD-treated HepG2 cells was much higher than that of the negative control without P-QD treatment (Figure S8). And a three-dimensional image reconstruction by TPM indicates that P-QD was internalized into cells via non-specific pathways (Figure S9). Moreover, the bright field image confirms that the cells are viable throughout the imaging studies. And the maximum tissue penetration depth of P-QD was also studied. Intralipid was chosen as a tissue simulating phantom medium because of its similar scattering properties with the real tissues^41^,^42^ (Supporting Information). As shown in Figure 3c, with the depth of tissue phantom increasing the luminescence signal from P-QD decreased drastically (see Figure S11 for more details). And the SBR-depth curve exhibited exponential profile (Figure 3d), similar to previous reports^42^,^43^. Note that the SBR of TP imaging was still higher than 1.7 even when the depth of tissue phantom was up to 2200 μm. This appreciable value of SBR indicates that the TP imaging depth achieved by the bright P-QD could be higher than 2200 μm, which is far exceeding that of the conventional organic dyes and even larger than that of the NIR emitting CdTe QDs (∼ 2000 μm)^43^ and Nitrogen-Doped Graphene QDs (∼ 1800 μm)^41^. For comparison, the TP imaging depth of Green Fluorescent Protein (GFP) was determined to be < 400 μm by a similar method (Figure S12).

**Figure 4:**
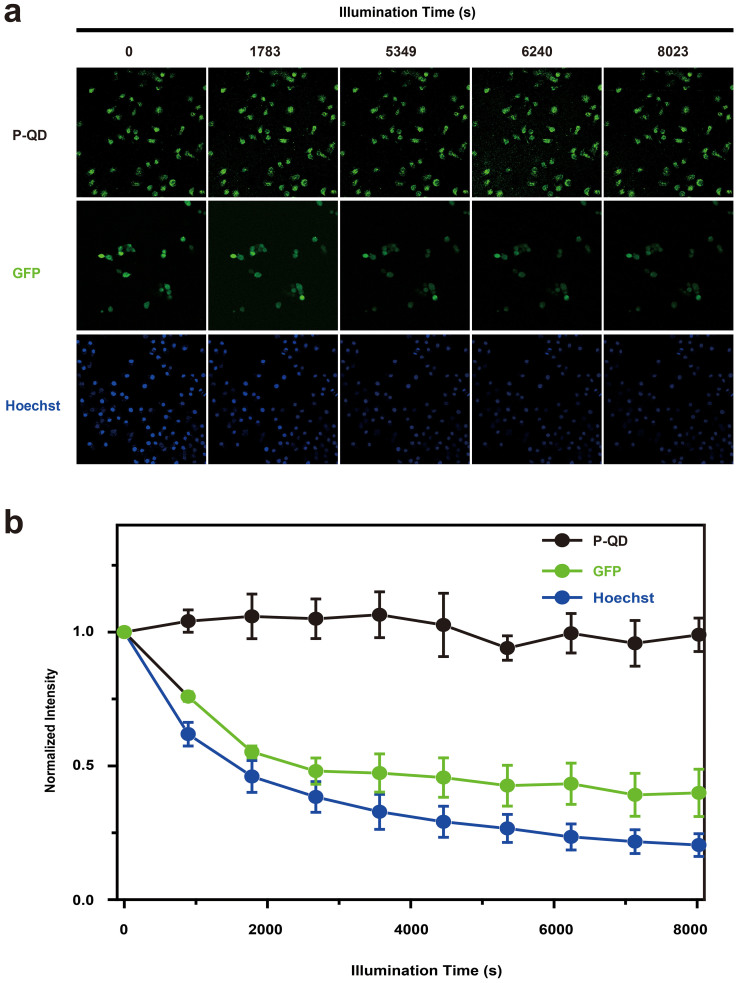
(a) Typical TP images of P-QD-treated HepG2 after different time intervals 800 nm femtosecond laser illumination. For comparison, the TP images of Hoechst 33342 and Green Fluorescent Protein (GFP) under the same conditions are shown. (b) The intensity of P-QD as a function of illumination time. For comparison, the fluorescence decay curves of Hoechst 33342 and Green Fluorescent Protein (GFP) under the same conditions are shown.

For a TP probe, due to the high photon flux used in TPM, photobleaching within the focal plane is an important issue. Although out-of-focus photobleaching minimized in TPM^3^, previous studies have indicated that dye molecules generally bleach more easily under TPE^44^ and some may even show higher order (> 2) photobleaching^45^ than with one-photon microscopy^46^,^47^. For our P-QD system, we interrogate the photostability with 800 femtosecond laser as excitation, with conventional dyes Hoechst 33342 and Green Fluorescent Protein (GFP) as control. As shown in Figure 4, P-QD within fixed cells maintained strong luminescent even after 8900 s femtosecond laser illumination and no apparent decrease of the emission signal was observed. In contrast, under the same conditions, Hoechst 33342 and GFP decreased ~ 80% and ~ 60%, respectively. Further study indicated that, in compared with the original QDs in THF, the photostability of P-QD was greatly enhanced (Figure S13), which can be ascribed to the shielding effect of polymer encapsulation^48^,^49^. Besides photostability, P-QD also exhibited strong resistance against pH- and ionic strength in the physiological region of pH 7 to 9 and ionic strength ~ 100 mM (Figure S10). We attribute this robust physiological environment stability to the plentiful surface-covered carboxyl groups, which acts as a protective shell around the nanoparticle^50^.

## Conclusion

To conclude, we have introduced a new class of polymer-encapsulated QDs with high TPE brightness σ × ϕ as high as 6.2 × 10^6^ GM (1 GM = 10^−50^ cm^4^ sphoton^−1^ particle^−1^). To our knowledge, the P-QD is current state-of-the-art TPA materials. And high signal-to-background cellular and tissue TP imaging with a penetration depth of 2200 μm were demonstrated with P-QD as probe. Due to its relative big size, the P-QD might not be appropriate for labeling fine cell structures, but these enhanced optical properties together with excellent biocompatibility make P-QD ideal for other bioimaging applications, such as single-molecule tracking and *in vivo* TP imaging. Additionally, as a robust material, we envision that the P-QD can also be used in quantitative biological studies, such as quantitative imaging and *in vivo* diagnostics^51^,^52^,^53^. Moreover, as our strategy is based on the universal hydrophobic interaction between non-polar species, we expect that the strategy developed here can easily be extended to other types of hydrophobic nanoparticles, such as near-infrared (NIR)-emitting QDs and magnetic nanoparticles, and that the as-prepared nanoparticles will find widespread application in nanobiotechnology.

## Methods

### Materials

Poly(methyl methacrylate-co-methacrylic acid) (PMMA-co-MAA), Mw: 34,000, molar proportion of methyl methacrylate to methacrylic acid is 1:0.016) was purchased from Sigma-Aldrich Corporation. Phosphate buffer saline (PBS, pH 7.4) solution was purchased from Beijing Solarbio Technology Corporation. All other chemicals of analytical grade were used as received. All aqueous solutions were prepared using deionized water with the conductivity of 18 MΩ cm.

### Synthesis of CdSeS/ZnS QDs

The CdSeS/ZnS QDs used were prepared according to the literature^54^,^55^ with minor modifications. Briefly, CdO (0.05 g), oleic acid (OA, 0.46 g), and tri-n-octylamine (TOA, 15 mL) were mixed in a three-neck round-bottom flask and heated to 300°C to get a clear solution under argon. A stock solution of Se (0.0021 g) and S (0.0124 g) in trioctylphosphine (1.0 mL) was swiftly injected into the hot solution of CdO/OA/TOA and allowed the reaction to proceed at 280°C for 1 min. The system was cooled down to 150°C and then heated to 240°C, while the mixture of ZnO (0.5 mmol) in OA stock solution (1.0 mL) and S (0.5 mmol) TOP solution (1.0 mL) was titrated into the flask and allowed the reaction proceed at 240°C for 1 minute. The CdSeS/ZnS QDs were obtained via precipitation with ethanol, washed, and dried in a vacuum oven for further studies.

### Preparation of P-QD

P-QD was prepared by our previously developed co-precipitation-assembly method. Typically, a THF solution (1.0 mL) containing CdSeS/ZnS QDs (0.5 mg) and PMMA-co-MAA (0.5 mg) was added dropwise into water (10.0 mL) under stirring (~ 100 rpm) at room temperature. The mixture was stirred for about 10 min, yielding a transparent colloidal solution. The THF in the colloidal solution was removed at 45°C by vacuum-rotary evaporation. Then P-QD were obtained via centrifugation at 50,000 g and re-dispersed under ultrasonic conditions in 10.0 mL of pure water.

### Cytotoxicity of P-QD

The cytotoxicity measurements were performed using MTT (3-(4,5-dimethylthiazol-2-yl)-2,5-diphenyltetrazolium bromide) assay. Briefly, HeLa and L929 cells were trypsinized and resuspended in Dulbecco’s modified Eagle’s medium (DMEM) containing 10% fetal bovine serum (FBS) and 80 U mL^−1^ gentamycin sulfate. The cells were seeded at a density of 0.2–1.0 million cells per well in a 96-well plate. After 24 h of incubation at 37°C in 5% CO_2_, the cells were washed 3 times with PBS solution (pH 7.4). Colloidal solutions of P-QD with different concentrations (100 μL) were added to the wells. After 12 h of incubation at 37°C in 5% CO_2_, a solution of MTT (20 μL, 5.0 mg mL^−1^) was added to each well. After 4 h of incubation at 37°C in 5% CO_2_, the medium was discarded, and the intracellular precipitate of formazan was collected by DMSO (100 μL). The absorbance at 570 nm was measured on a BioRad Model 550 microplate reader. Each data point was collected by averaging that of six wells, and the untreated cells were used as controls.

### Live-cell TP imaging

HepG2 cells were propagated in DMEM supplemented with 10% fetal bovine serum (FBS) and 80 U mL^−1^ gentamycin sulfate. Then the cultured cells were trypsinized and re-suspended in this DMEM Medium at a concentration of about 7.5 × 10^5^ mL^−1^. The cell suspension (100 μL) was transferred to a confocal dish (35 mm). After incubation for 24 h at 37°C in 5% CO_2_, the cells were carefully rinsed with PBS solution (pH 7.4). Then a colloidal solution of P-QD (100 μL, 0.1 mg mL^−1^) was added. After 2 h incubation at 37°C in 5% CO_2_, the dish was rinsed three times with PBS solution (pH 7.4) and then 1 mL of fresh serum-free medium was added. The plates were incubated for another 10 minutes at 37°C and then directly imaged on a Zeiss LSM 780 confocal microscope system equipment with Spectra-physics MaiTai Ti:sapphire laser (100 fs, 80 MHz repetition rate) and a water objective lens (20 ×). The excitation wavelength was 800 nm fs laser, while emission was collected through a 500 to 550 nm band-pass filter with non-descanned detectors (NDD) PMT detector. For comparison, all of the TP images were taken at the same setting. And the bright field images were taken on the same instrument. ZEN 2012 software was used for capturing and processing images.

### Two-photon deep-tissue imaging

Deep-tissue imaging were performed with Intralipid as tissue simulating phantom medium. The depth of tissue phantom was carefully varied with the volume of Intralipid. And the excitation wavelength was 800 nm fs laser, and the full output power corresponding to approximately 180 mW average power was used to achieve the fluorescence image of HepG2 cells in the turbid tissue phantom.

## Author Contributions

Y.Y.F., R.C.H. and Y.Q.J. designed the experiment. Y.Y.F., R.C.H., and H.L.L. conducted all experiments. R.C.H. wrote the paper. L.H. and H.S. assisted with the two-photon imaging. R.C.H., Y.L.S. and Y.Q.J. commented on the manuscript at all stages. All the authors contributed on the discussion part of all the results.

## Supplementary Material

Supplementary Information Extremely High Brightness from Polymer-Encapsulated Quantum Dots for Two-photon Cellular and Deep-tissue Imaging

## Figures and Tables

**Figure 1:**
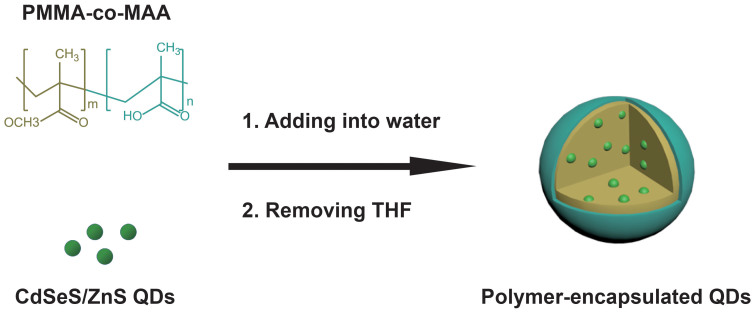
Schematic illustration of the preparation route for the P-QD using PMMA-co-MAA polymers.
