# Supplementary material for: Extremely High Brightness from Polymer-Encapsulated Quantum Dots for Two-photon Cellular and Deep-tissue Imaging
Source: Sci Rep. 2015 Apr 24;5:9908. doi: 10.1038/srep09908 (PMC4408994; doi:10.1038/srep09908)
Supplement: Supplementary Information — Extremely High Brightness from Polymer-Encapsulated Quantum Dots for Two-photon Cellular and Deep-tissue Imaging [file srep09908-s1.pdf]

Supporting Information

Extremely High Brightness from Polymer-Encapsulated Quantum Dots for Two-photon Cellular and Deep-tissue Imaging

Yanyan Fan,[†, §] *Helin Liu,*[‡] *Rongcheng Han,*[*,†] *Lu Huang,*[†] *Hao Shi,*[†] *Yinlin Sha,*[*,‡] *and Yuqiang Jiang*[*,†]

[†] State Key Laboratory of Molecular Developmental Biology, Institute of Genetics and Developmental Biology, Chinese Academy of Sciences, Beijing 100101, China.

[‡] Department of Biophysics, School of Basic Medical Sciences and Biomed-X Center, Peking University, Beijing 100191, China.

[§] Institute of Opto-electronic Materials and Technology, South China Normal University, Guangzhou 510631, China.

Synthesis of CdSeS/ZnS QDs**:** The CdSeS/ZnS QDs used were prepared according to the literature[S1] with minor modifications. Briefly, CdO (0.05 g), oleic acid (OA, 0.46 g), and tri-n-octylamine (TOA, 15 mL) were mixed in a three-neck round-bottom flask and heated to 300 ℃ to get a clear solution under argon. A stock solution of Se (0.0021 g) and S (0.0124 g) in trioctylphosphine (1.0 mL) was swiftly injected into the hot solution of CdO/OA/TOA and allowed the reaction to proceed at 280 ℃ for 1 min. The system was cooled down to 150 ℃ and then heated to 240 ℃, while the mixture of ZnO (0.5 mmol) in OA stock solution (1.0 mL) and S (0.5 mmol) TOP solution (1.0 mL) was titrated into the flask and allowed the reaction proceed at 240 ℃ for 1 minute. The CdSeS/ZnS QDs were obtained via precipitation with ethanol, washed, and dried in a vacuum oven for further studies.

Fluorescence lifetime and photoluminescence quantum yield measurement**:** Fluorescence lifetime measurements were performed on a F900 Edinburgh Instruments spectrometer by the time correlated single photon counting (TCSPC) method. Measurements were taken in ambient conditions, at room temperature, on solutions diluted to yield reasonable signal intensity. Excitation was performed at 514 nm and the time-resolved emission data were collected at 530 nm. The TCSPC traces were analyzed by standard tail fit implemented in the software of the fluorimeter. Weighted residuals and χ^2 values were used to judge the quality of the fit.

The photoluminescence quantum yield (ϕ) of P-QD was determined according to the method described by Demas and Grosby[S2], using FITC (fluorescein isothiocyanate) (ϕ = 0.96) in basic aqueous solution (pH=11) as the reference. In the experiments, the absorption and PL emission spectra were acquired on a TU-1901 UV-Vis

spectrophotometer (Puxi Tongyong Co.) and an F-4500 fluorescence spectrophotometer (Hitachi), respectively. Multiple concentrations were examined and the integration area of PL emissions was plotted with the absorption intensity at each concentration (Figure S4). The data for a given sample with a given excitation wavelength but with different concentrations should fit nicely in a linear line that must go through the origin. The measurements were conducted at a temperature of 20 ℃, and the PL excitation wavelength was set at 468 nm. The PL QY was calculated using the following Equation:

$$\phi_{QDs} = \phi_{ref} \times \frac{k_{QDs}}{k_{ref}} \times \left(\frac{n_{ref}}{n_{QDs}}\right)^2$$

Ref: FITC; k: slope of absorption-emission curve; n: refractive index of a solvent (refractive index: water=1.333; THF =1.407).

To explore the exact mechanism behind our QY enhancement phenomena, the fluorescence lifetime of QDs and P-QD were measured. As shown in Figure S5, these kinetics decay traces were fitted by double exponential decay functions, which is typical for QDs emission.[S3,S4] The double exponential decay is described as:

$$I(\mathrm{t}) = A_1 \exp\left(\frac{t}{\tau_1}\right) + A_2 \exp\left(\frac{t}{\tau_2}\right)$$

where τ_1 and τ_2 are a shorter lifetime and a longer lifetime, respectively, and A_i represents an intensity of decay process with the lifetime of τ_i. The shorter lifetime of our samples was ~4 ns, which can be attributed to the excitonic states recombination[S5]. And the slow decay component of ~24 ns comes from the surface trapping state[S4, S6].

Further analyses reveal that the radiative decay rate (k_r) of P-QD was improved up to 6.63×10^7 s^{-1}, about 3.9-fold of the original CdSeS/ZnS QD (k_r, 1.71×10^7 s^{-1}), while the nonradiative decay rate exhibited a 2.8-fold decrease from 3.48×10^7 s^{-1} to 1.23×10^7 s^{-1} (Table S1). Together with the fractional weight changes (A_1 and A_2), it is reasonable to postulate that the QY improvement of P-QD is mainly from the efficiently blocking nonradiative decays pathway from surface traps.

Table S1. PL QY, time constants τ_1 and τ_2, the fractional weights of the various decay time components A_1 and A_2, average lifetime τ and the radiative/nonradiative decay rate of the the original CdSeS/ZnS QDs in THF and the as-prepared P-QD in water.

sample	QY	τ_1(ns)	A_1%	τ_2(ns)	A_2%	τ_m(ns)	k_r^* ($\times10^7 s^{-1}$)	k_{nr}^* ($\times10^7 s^{-1}$)
QDs in THF	0.33	4.67	25.63	24.3	74.37	19.3	1.71	3.48
P-QD (1:1)	0.84	2.31	40.71	19.9	59.26	12.7	6.63	1.23
P-QD (1:2)	0.83	-	-	-	-	-	-	-
P-QD (1:0.2)	0.46	4.09	45.1	21.6	54.9	13.7	5.27	2.03
P-QD (1:0.1)	0.38	4.02	46.31	21.6	53.69	13.5	5.11	2.33
P-QD (1:5)	0.72	5.75	36.75	25.8	63.25	18.4	2.50	2.93
P-QD (1:10)	0.68	5.20	31.65	27.2	68.35	20.2	1.88	3.07

* QY= $k_r/(k_r+k_{nr}) = k_r\tau$, where k_r, k_{nr}, and τ stand for the radiative decay rate, the nonradiative decay rate, and the mean luminescence lifetime, respectively. Therefore, k_r could be calculated k_r=QY/τ ; $K_{nr}=1/\tau- k_r$.

Estimated TP excitation action cross-section of P-QD: The concentration of QDs (0.1 mg mL^{-1}) was determined to be 139.7 nM according to the literature[S7]. And the TPE brightness of QDs $(\sigma \times \phi)_{QDs}$ in THF was determined to be 3.1 $\times 10^3$ GM, which is consistent with previous reports [S8, S9]. The diameter of QDs and P-QD was determined to be 4 nm and 27 nm, respectively. And thus the estimated numbers of QDs in a P-QD was $N = (d_{P\text{-}QD}/d_{QDs})^3 = (27/4)^3 = 307$. Therefore, the TPE brightness of P-QD $(\sigma \times \phi)_{P\text{-}QD}$ was calculated as $N * (\sigma \times \phi)_{QDs} * \Phi_{P\text{-}QD}/\Phi_{QDs} = 307*2.6*3.1\times 10^3$ GM$=6.2\times 10^6$ GM.

Table S2. The TPA cross-sections (σ) and the TPE brightness σ × ϕ of the original CdSeS/ZnS QDs in THF and the as-prepared P-QD in water.

	**σ**		**σ × ϕ**	
**λ (nm)**	**QDs**	**P-QD**	**QDs**	**P-QD**
**720**	8.70E+03	6.84E+06	2.86E+03	5.74E+06
**730**	8.18E+03	6.43E+06	2.70E+03	5.40E+06
**740**	4.13E+03	3.24E+06	1.36E+03	2.72E+06
**750**	5.40E+03	4.24E+06	1.78E+03	3.56E+06
**760**	6.20E+03	4.88E+06	2.04E+03	4.10E+06
**770**	5.98E+03	4.70E+06	1.97E+03	3.95E+06
**780**	5.87E+03	4.61E+06	1.93E+03	3.87E+06
**790**	8.48E+03	6.66E+06	2.79E+03	5.60E+06
**800**	9.36E+03	7.36E+06	3.08E+03	6.18E+06
**810**	7.58E+03	5.96E+06	2.50E+03	5.00E+06
**820**	7.20E+03	5.66E+06	2.37E+03	4.75E+06
**830**	6.48E+03	5.09E+06	2.13E+03	4.28E+06
**840**	5.99E+03	4.71E+06	1.97E+03	3.95E+06
**850**	5.48E+03	4.30E+06	1.80E+03	3.62E+06
**860**	3.32E+03	2.61E+06	1.09E+03	2.19E+06
**870**	2.63E+03	2.07E+06	8.68E+02	1.74E+06
**880**	1.57E+03	1.23E+06	5.16E+02	1.03E+06

Investigation of P-QD Stability: We further interrogate the pH- and ionic strength-stability in the physiological region of pH 7 to 9 and ionic strength ~100 mM. Notably, P-QD in water maintain strong PL in the wide pH range of 5-12 (Figure S10) with varying less than 5% (normalized by PL intensity at pH 7), although QDs significantly quenched at pH 2 (>97%) and pH 13 (>70%). These results are consistent with previous report, in which silica and amphiphilic polymer were used to stabilize CdSe/ZnS QDs and remarkable stability was achieved[S10]. Previous studies have shown QDs significantly quench at low pH values as a result of detachment of the surface ligand and subsequent the formation of aggregates or the increased defect-related surface states[S11]. As for the decrease in PL intensity of P-QD under basic conditions, it might be associated with partial degradation of the PMMA-co-MAA shell in strong bases and thus QDs become sensitive to environment again. In different ionic strength solution, P-QD present high luminescence even when the concentration of NaCL is up to 200 mM and there is still not any precipitation.

Photobleaching Test: For photobleaching test, P-QD-treated HepG2 cells in PBS were exposed with 800 nm fs laser for 8000 s, and conventional dyes Hoechst 33342 and Green Fluorescent Protein (GFP) as control. And before photobleaching test, all cells were fixed with paraformaldehyde.

Two-Photon Deep-Tissue Imaging: Deep-tissue imaging was performed with Intralipid as tissue simulating phantom medium. The depth of tissue phantom was carefully varied with the volume of Intralipid. And the excitation wavelength was 800

nm fs laser, and the full output power corresponding to approximately 180 mW average power was used to achieve the fluorescence image of HepG2 cells in the turbid tissue phantom.

Intralipid was chosen as a tissue simulating phantom medium because of its similar scattering properties with the real tissues[S12]. For further optical information, such as the scattering coefficient (μs) and anisotropy (g), of the intralipid simulating tissue phantom, please read the literature[S13].

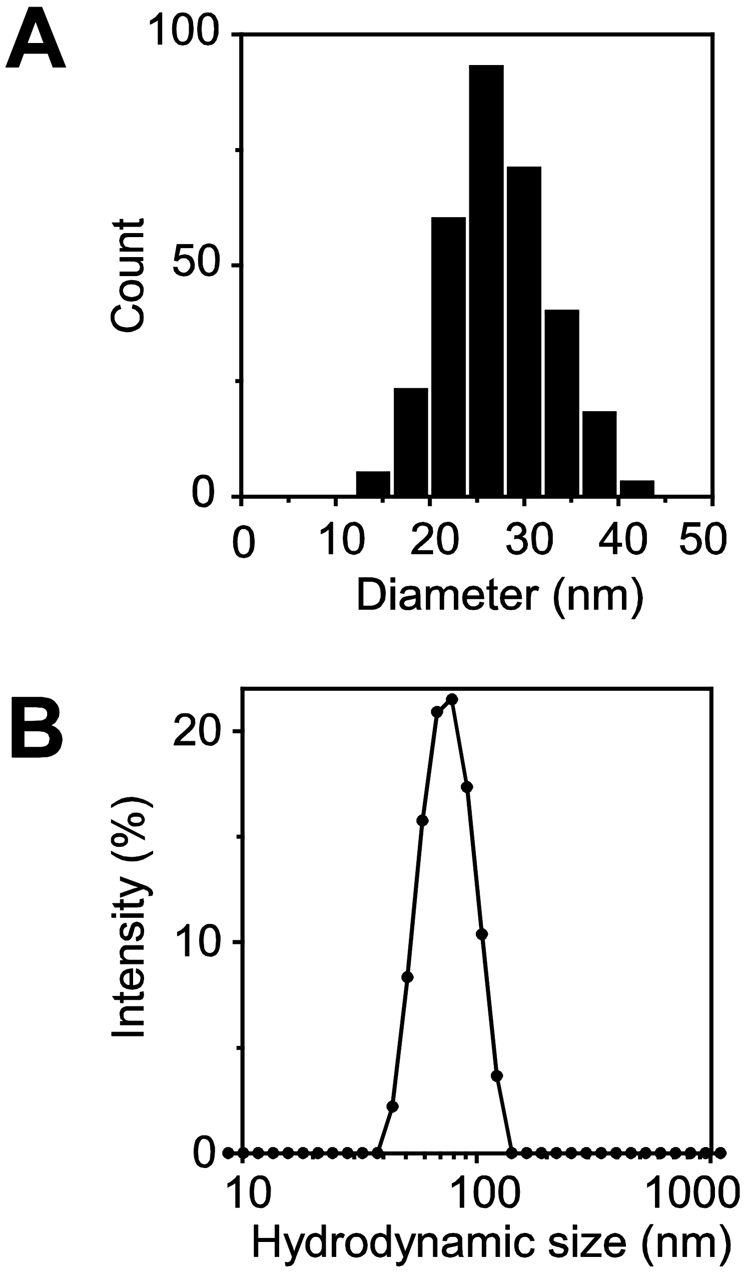

Figure S1. (a) The size distribution of P-QD determined by TEM. (b) The corresponding hydrodynamic size of the P-QD.

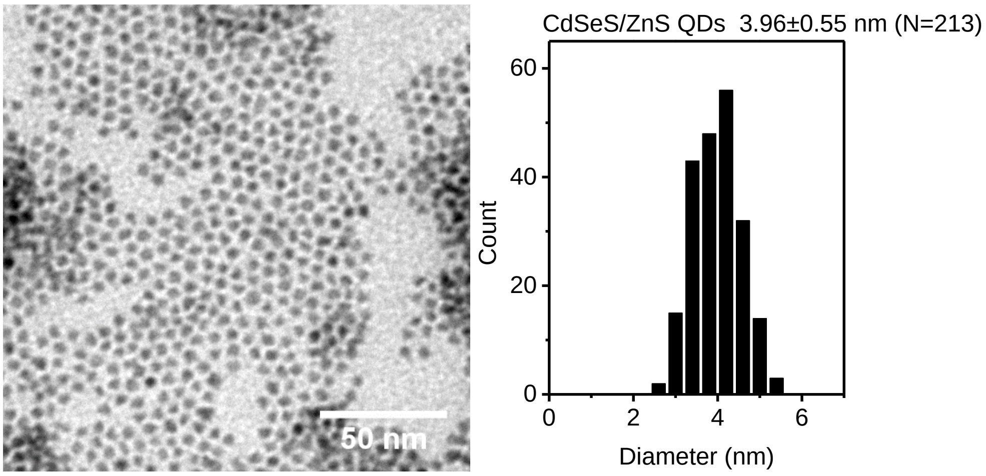

Figure S2. TEM of original CdSeS/ZnS QDs and the corresponding size distribution.

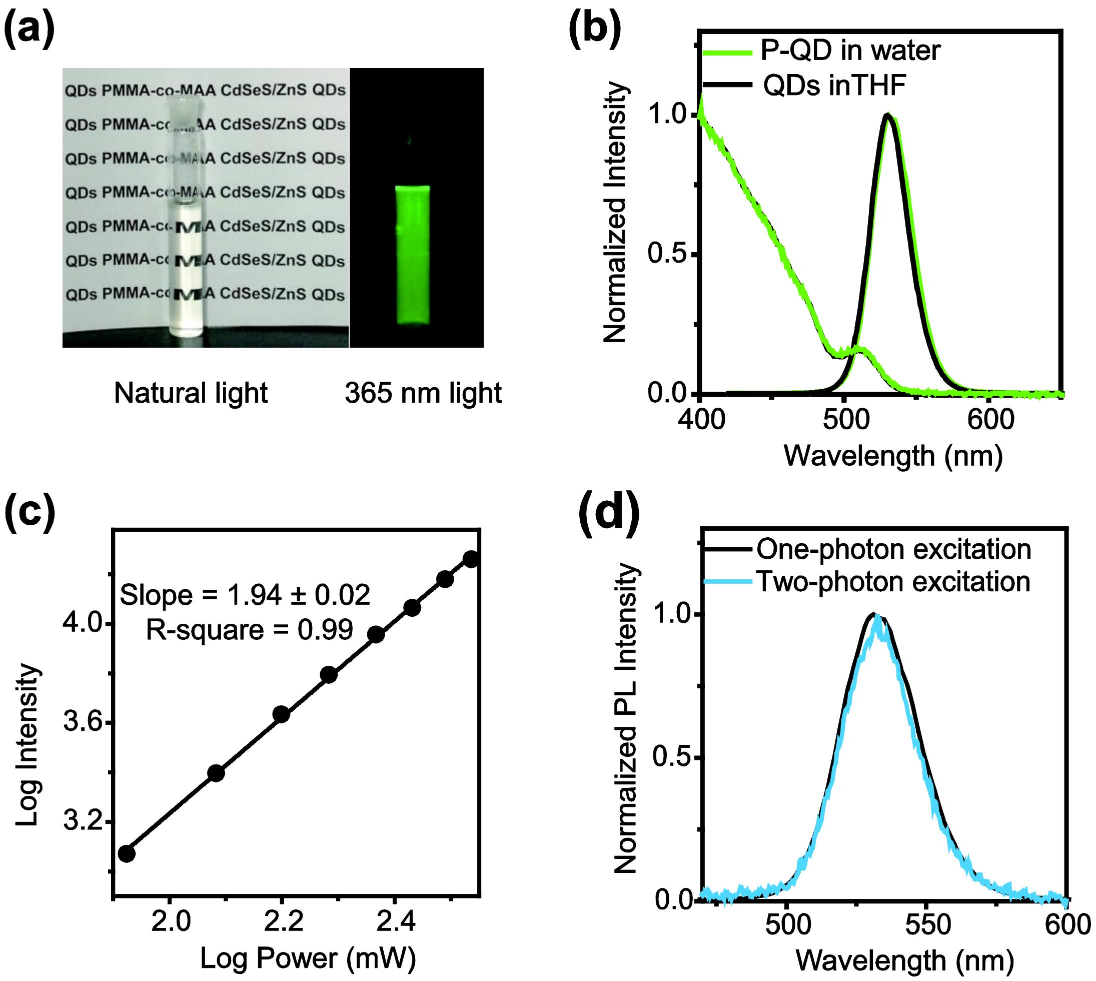

Figure S3. (a) Photograph of aqueous solution of P-QD taken under 365 nm UV lamp irradiation. (b) UV-vis absorption and one-photon excitation photoluminescence

spectra (λ_{ex}= 400 nm) of CdSeS/ZnS QDs in THF and P-QD in water. (c) Quadratic relationship of the luminescence intensity of the P-QD aqueous solution with various excitation laser powers at 800 nm (λ_{em}= 530 nm). (d) TP excitation luminescence spectrum of P-QD under 800 nm femtosecond laser excitation and one-photon excitation luminescence spectrum of P-QD (λ_{ex}= 400 nm).

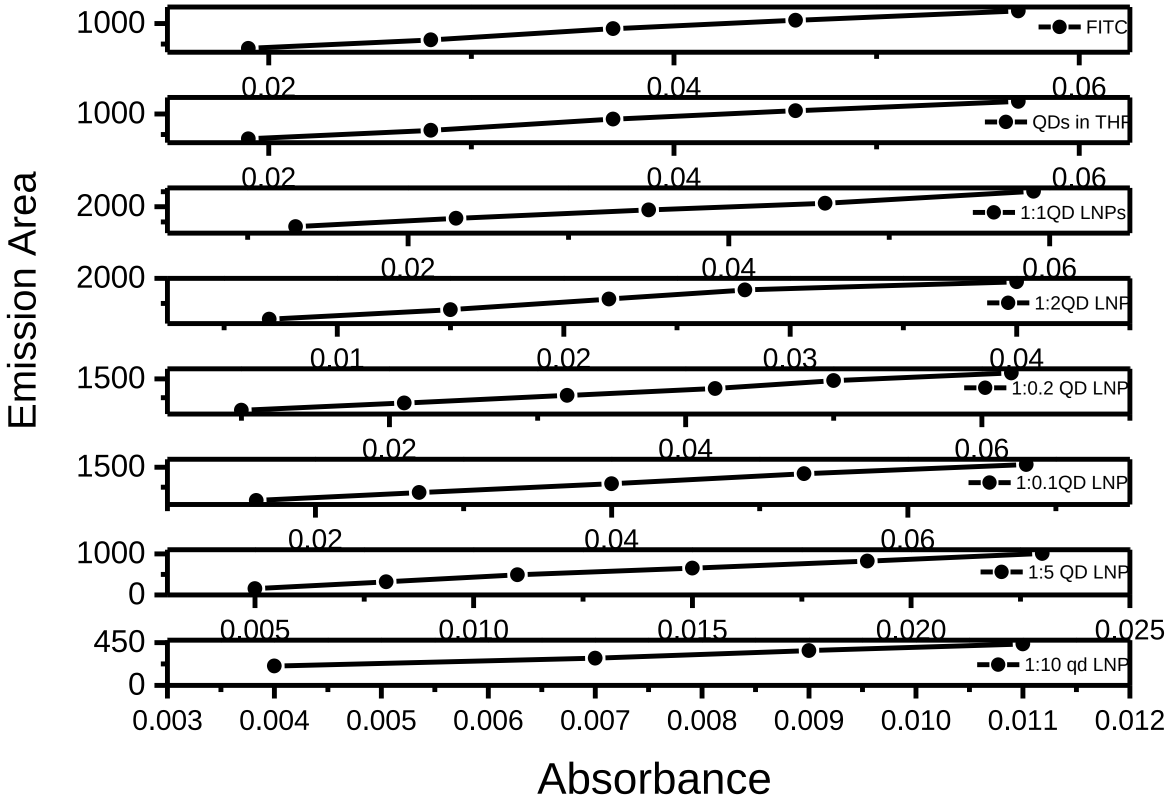

Figure S4. The QY measurement of P-QD with various ratios of QDs and PMMA-co-MAA. For comparison, the curve of FITC and QDs in THF were also shown as reference.

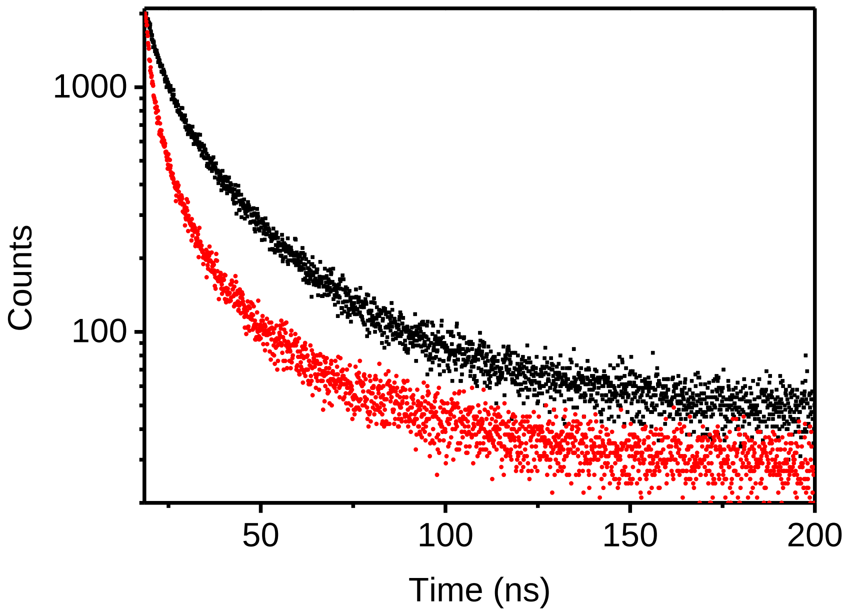

Figure S5. Representative luminescence decays for the original CdSeS/ZnS QDs in THF (black) and the as-prepared P-QD in water (red).

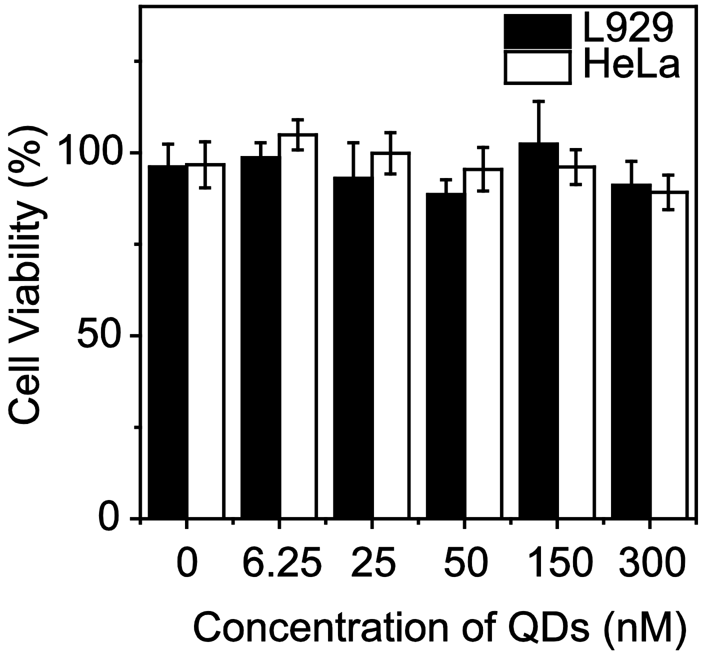

Figure S6. Viability of L929 and HeLa cells in the presence of P-QD as determined by MTT assay. Data are represented as mean ± SD (n=6). The untreated cells were used as the control.

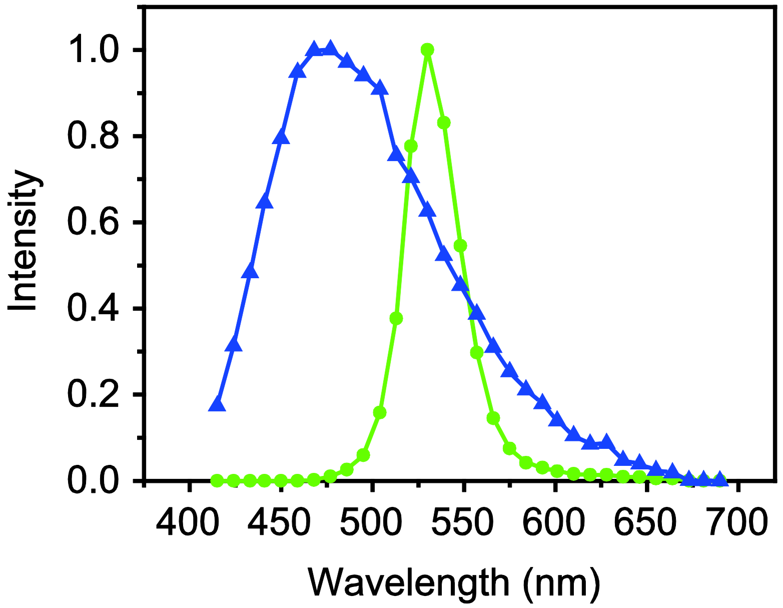

Figure S7. The emission profiles after linear unmixing according to the corresponding spectral images in Figure 3a.

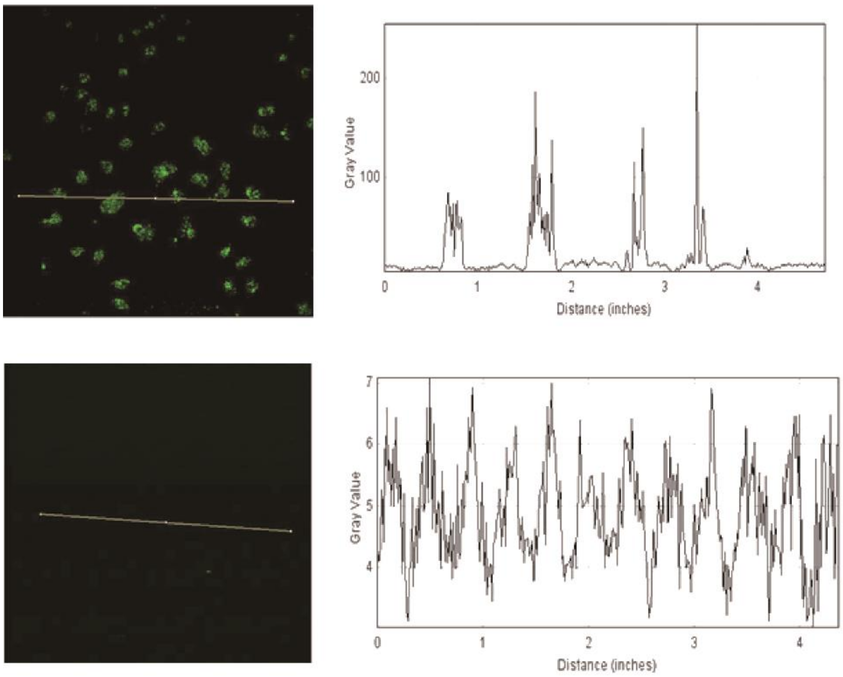

Figure S8. Bright TPE signal was detected from P-QD-treated HepG2 cells (upper panel), while little signal from the negative control (lower panel) without P-QD treatment. Notably the signal-to-background ratio (SBR) in P-QD-treated HepG2 cells was extremely higher than that in the negative control.

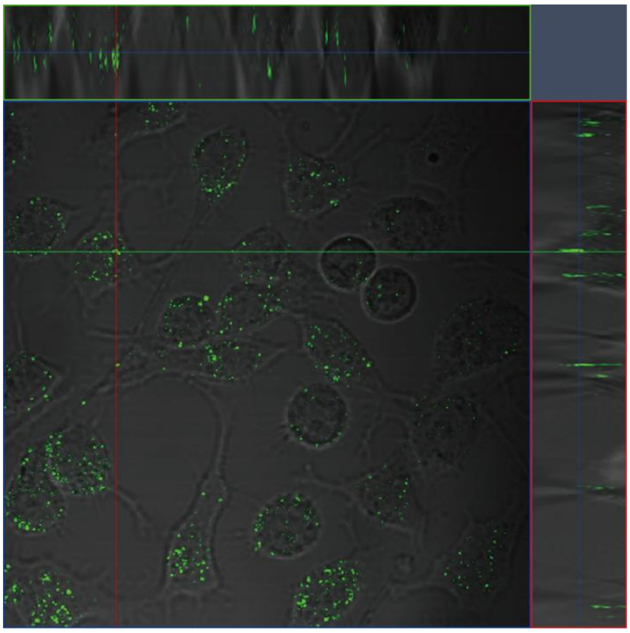

Figure S9. Orthogonal view 3D TPM of P-QD-treated HepG2 cells. The excitation wavelength was 800 nm fs laser, while emission was collected through a 500 to 550 nm band-pass filter with non-descanned detectors (NDD) PMT detector.

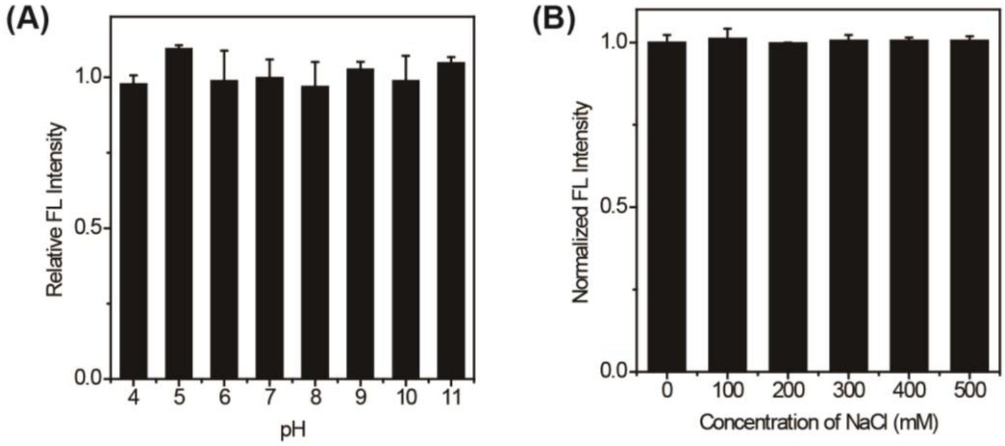

Figure S10. The pH- and ionic strength-stability of P-QD evaluated under various pH

and ionic strength.

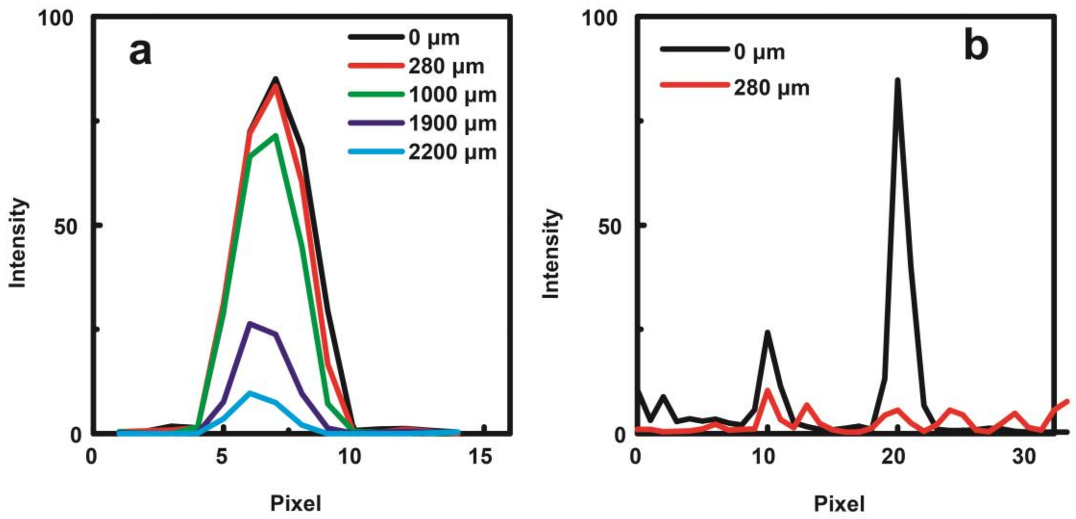

Figure S11. Representative line profiles of P-QD in tissue phantom under (a) two-photon excitation and one-photon excitation with increased depth.

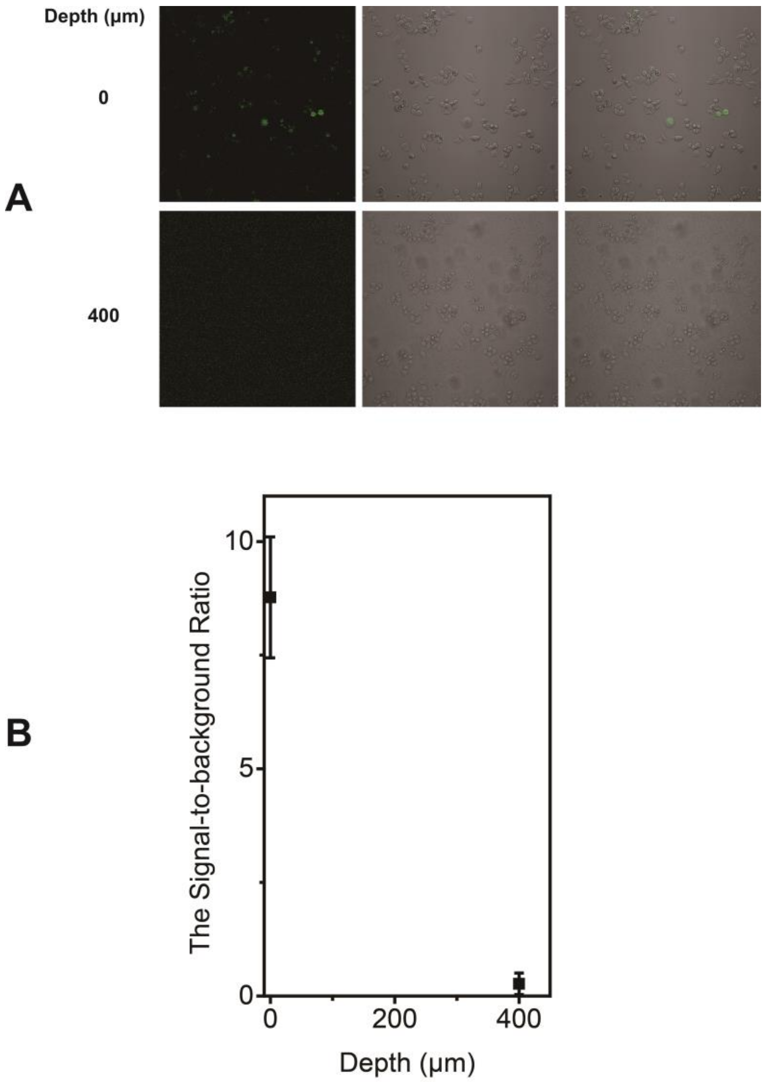

Figure S12. (a) Two-photon imaging of HeLa cells that partially express green fluorescent protein (GFP), 800 nm femtosecond laser as excitation. Left: Fluorescence; middle: DIC; right: overlay. (b) Imaging depth of GFP in tissue phantom under 800 nm femtosecond laser excitation. Left: TPE; middle: DIC; right: Overlay. (d) The signal-to-background ratio (SBR) as a function of depth. SBR was defined as the ratio of ($signal_{with\ GFP}$ - $signal_{without\ GFP}$) to $signal_{without\ GFP}$.

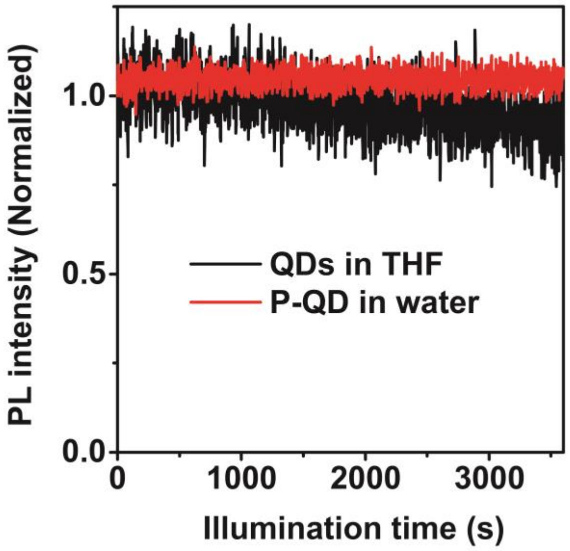

Figure S13. Time courses of PL intensity changes of P-QD in water and QDs in THF under the continuous illumination. The experiments were performed on an F-4500 fluorescence spectrophotometer (Hitachi，Japan) with Time Scan Mode (λ_{ex} = 400 nm, λ_{em} = 530 nm). The concentration of QDs and P-QD was tuned to be same with an absorption of 0.03. The split widths of excitation and emission were set at 2.5 and 2.5 nm, respectively. No extra protection against O_2 was conducted.

References

[S1] a) E. Jang, S. Jun, L. Pu, *Chem. Commun.* **2003**, *82*, 2964; b) R. C. Han, M. Yu, Q. Zheng, L. J. Wang, Y. K. Hong, Y. L. Sha, *Langmuir* **2009**, *25*, 12250.

[S2] J. N. Demas, G. A. Crosby, *J. Phys. Chem-US* **1971**, *75*, 991.

[S3] B. R. Fisher, H. J. Eisler, N. E. Stott, M. G. Bawendi, *J. Phys. Chem. B* **2004**, *108*, 143.

[S4] X. Y. Wang, L. H. Qu, J. Y. Zhang, X. G. Peng, M. Xiao, *Nano Lett.* **2003**, *3*, 1103.

[S5] a) V. I. Klimov, D. W. McBranch, C. A. Leatherdale, M. G. Bawendi, *Phys. Rev. B* **1999**, *60*, 13740; b) M. G. Bawendi, P. J. Carroll, W. L. Wilson, L. E. Brus, *J. Chem. Phys.* **1992**, *96*, 946.

[S6] V. I. Klimov, *J. Phys. Chem. B* **2000**, *104*, 6112.

[S7] W. W. Yu, L. H. Qu, W. Z. Guo, X. G. Peng, *Chem. Mater.* **2003**, *15*, 2854.

[S8] D. R. Larson, W. R. Zipfel, R. M. Williams, S. W. Clark, M. P. Bruchez, F. W. Wise, W. W. Webb, *Science* **2003**, *300*, 1434.

[S9] a) L. Zhao, F. Wu, W. Tian, C. Li, *Acta Opt. Sin.* **2009**, *29*, 1332; b) W. Feng, T. Wei, M. Li-Na, C. Wen-Ju, Z. Gui-Lan, Z. Guo-Feng, C. Shi-Dong, X. Wei, *Chinese Phys. Lett.* **2008**, *25*, 1461.

[S10] Hu, X. G. & Gao, X. H. Silica-Polymer Dual Layer-Encapsulated Quantum Dots with Remarkable Stability. *ACS Nano* **2010**, *4*, 6080.

[S11]a) Y. S. Liu, Y. H. Sun, P. T. Vernier, C. H. Liang, S. Y. C. Chong, M. A. Gundersen, *J. Phys. Chem. C* **2007**, *111*, 2872; b) A. Mandal, N. Tamai, *J. Phys. Chem. C* **2008**, 112, 8244.

[S12] a) Liu, Q., Guo, B. D., Rao, Z. Y., Zhang, B. H. & Gong, J. R. Strong Two-Photon-Induced Fluorescence from Photostable, Biocompatible Nitrogen-Doped Graphene Quantum Dots for Cellular and Deep-Tissue Imaging. *Nano Lett* **2013**, *13*, 2436. b) Welsher, K., Sherlock, S. P. & Dai, H. J. Deep-tissue anatomical imaging of mice using carbon nanotube fluorophores in the second near-infrared window. *P Natl Acad Sci USA* **2011**,*108*, 8943.

[S13] a) Flock, S. T., Jacques, S. L., Wilson, B. C., Star, W. M. & Vangemert, M. J. C. Optical-Properties of Intralipid - a Phantom Medium for Light-Propagation Studies. *Laser Surg Med* **1992**, *12*, 510. b) Driver, I., Feather, J. W., King, P. R. & Dawson, J. B. The Optical-Properties of Aqueous Suspensions of Intralipid, a Fat Emulsion. *Phys Med Biol* **1998**, *34*, 1927. c) Assadi, H., Karshafian, R. & Douplik, A. Optical Scattering Properties of Intralipid Phantom in Presence of Encapsulated Microbubbles. *International Journal of Photoenergy*, **2014**, Article ID 471764, doi:10.1155/2014/471764. d) Vanstaveren, H. J., Moes, C. J. M., Vanmarle, J., Prahl, S. A. & Vangemert, M. J. C. Light-Scattering in Intralipid-10-Percent in the Wavelength Range of 400-1100 nm. *Appl Optics* **1991**, *30*, 4507.
